# Association between low back pain perception and occupational in hospital nursing professionals

**DOI:** 10.47626/1679-4435-2022-1012

**Published:** 2024-09-24

**Authors:** Dionata Cristiano Schmidt, Tiago da Rosa Rambo, Eduarda Chaves Silveira, Litiele Evelin Wagner, Jane Dagmar Pollo Renner, Dulciane Nunes Paiva

**Affiliations:** 1 Stricto Sensu Graduate Program Master’s and Doctorate in Health Promotion, Universidade de Santa Cruz do Sul (UNISC), Santa Cruz do Sul, RS, Brazil; 2 School of Physical Therapy, UNISC, Santa Cruz do Sul, RS, Brazil; 3 Stricto Sensu Gradute Program in Respiratory Sciences, Universidade Federal do Rio Grande do Sul, Porto Alegre, RS, Brazil; 4 Life Sciences Department, UNISC, Santa Cruz do Sul, RS, Brazil; 5 Health Sciences Department, UNISC, Santa Cruz do Sul, RS, Brazil

**Keywords:** low back pain, nursing staff, occupational stress, dor lombar, equipe de enfermagem, estresse ocupacional

## Abstract

**Introduction:**

Low back pain can be defined as pain and/or discomfort between the coastal margins of the lowest rib and the gluteal fold, and it can cause motor dysfunction, loss of productivity, and changes in job function. There is a greater number of nursing professionals in hospital environments, and, regardless of their numerical contingent, they have the highest percentage of absenteeism due to this condition.

**Objectives:**

To evaluate the association between perception of low back pain and occupational stress in hospital nursing professionals.

**Methods:**

Eleven nurses and 95 practical nurses (n = 106) were evaluated on their perception of low back pain (Visual Analogue Scale) and occupational stress (job stress scale) using the Demand-Control Model. The chi-square test was used to assess associations between categorial variables (p < 0.05).

**Results:**

Low back pain was reported by 74% of the study sample (n = 81). Assessment of occupational stress using the Demand-Control Model showed that 54.7% (n = 58) had low psychological demand and 63.2% (n = 67) had high control at work. Active work was observed in 33% (n = 35) and low demand at work in 30.2% (n = 32). There were no significantly associations between the perception of low back pain and the occupational stress domains described by the Demand-Control Model (p = 0.721).

**Conclusions:**

Although there was a high prevalence of low back pain in this sample of nursing professionals, it was not associated with occupational stress.

## Abstract (pt)

**Introdução:**

A dor lombar pode ser definida como dor e/ou desconforto entre as margens costais da última costela e a prega glútea, podendo causar disfunções motoras, perda de produtividade e mudança de emprego. Os profissionais de enfermagem atuam em maior número no âmbito hospitalar e, independentemente de seu contingente numérico, apresentam o maior percentual de absenteísmo devido a essa afecção.

**Objetivos:**

Avaliar a associação entre a percepção de dor lombar e o estresse ocupacional em profissionais de enfermagem de âmbito hospitalar.

**Métodos:**

Foram avaliados 11 enfermeiros e 95 técnicos de enfermagem (n = 106) quanto à percepção da dor lombar (Escala Visual Analógica) e o estresse ocupacional ( Job Stress Scale) por meio do Modelo Demanda-Controle. A associação entre variáveis categóricas foi avaliada pelo teste de qui-quadrado (p < 0,05).

**Resultados:**

Houve prevalência de dor lombar em 74% da amostra (n = 81). A avaliação do estresse ocupacional por meio do Modelo Demanda-Controle evidenciou que 54,7% (n = 58) apresentou baixa demanda psicológica e 63,2% (n = 67) apresentou alto controle no trabalho. O trabalho ativo foi evidenciado em 33% (n = 35) e a baixa exigência no trabalho em 30,2% (n = 32). Não houve associação entre a percepção de dor lombar e os domínios do estresse ocupacional (p = 0,721).

**Conclusões:**

Houve alta prevalência de dor lombar, sem que tal sintoma tenha se associado ao estresse ocupacional nos profissionais de enfermagem avaliados.

## INTRODUCTION

Low back pain (LBP) was reported by the 2010 Global Burden of Disease (GBD) as one of the 10 main diseases caused by high-burden injuries,^1^ defined as pain and/or discomfort between the coastal margins of the lowest rib and the gluteal fold. In addition to causing motor dysfunctions, it can lead to loss of productivity, and those affected might even have to change jobs.^2^ Nursing professionals predominantly work in hospital settings and have the highest rates of absenteeism due to LBP. Although this condition is one of the most common causes of musculoskeletal disorders and is responsible for severe disabilities, it is often overlooked.^3,4,5^

In the hospital setting, LBP reduces productivity and the quality of care provided to hospitalized patients,^6^ which can cause a lack of job satisfaction and high costs to the health care system.^7^ The nursing profession involves intense physical effort during handling of patients and equipment, which can lead to muscle pain and/or discomfort, contributing to the reduction of strength and work capacity.^5^ Biomechanical studies indicate that this professional category has a higher risk of developing LBP, as the strenuous activities they perform can overstrain the spinal column.^8^ In addition to physical and behavioral aspects, risk factors also include workplace conditions, workload, stress, and job satisfaction.^9^

Occupational stress is related to negative stimuli in the work process/environment, which have deleterious effects on the workers’ physical and/or psychological health.^10^ Individual and behavioral characteristics also affect the occupational environment. Therefore, conflicting environments with excessive physical and psychological strain can elevate stress levels, exacerbating physical and emotional impairment and reducing productivity and well-being.^11^ In this sense, work activities performed by nursing professionals can generate unfavorable indicators of physiological and/or psychological stress, which may significantly change some aspects of social life and the family environment.^12^

Some studies have evaluated occupational stress^13,14^ and the presence of LBP^11,15^ in nursing professionals. In Saudi Arabia, the association between occupational stress and the presence of LBP in this population has been investigated.^7^ However, it is still unclear how these variables correlate when considering the specificities of health care services in Brazil. Brazil operates under a universal health care system but with reduced government investment, leading to inequity, overload on health care professionals, and exacerbation of imbalances in the labor market.^15,16^

LBP needs to be further explored as an occupational problem related to stress. The importance of this topic can only be understood when the full impact of LBP on the quality of life of professionals who need to provide high-quality care for hospitalized patients is acknowledged. Therefore, this study aimed to evaluate the association between the perception of LBP and occupational stress in hospital nursing professionals.

## METHODS

**Figure 1:**
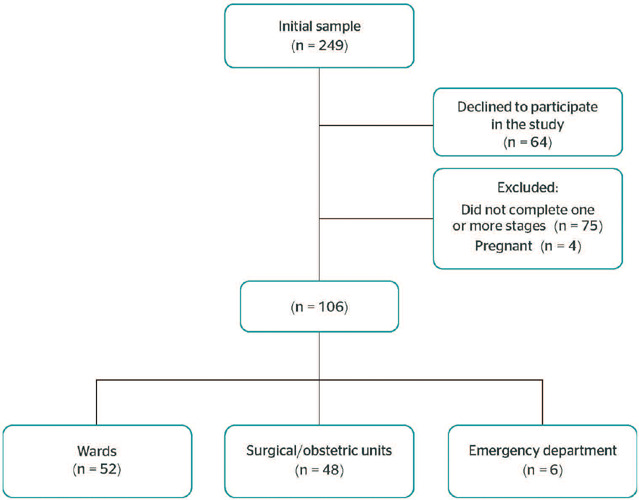
Study flowchart.

This was a cross-sectional study including 249 nursing professionals, of whom 62 were nurses and 187 were practical nurses. Of the total number of participants, 64 declined to participate, and 75 were excluded for not completing one or more stages of the study. An additional 4 participants were excluded because they were pregnant. Thus, the final sample consisted of 106 eligible professionals (11 nurses and 95 practical nurses) according to the study’s inclusion criteria, all of whom were affiliated with a university hospital located in the city of Santa Cruz do Sul, state of Rio Grande do Sul, Brazil (Figure 1).

The study population was recruited via nonprobabilistic convenience sampling from April to September 2017. Male and female nursing professionals, aged between 18 and 60 years, who were affiliated with the hospital institution in accordance with labor laws were included. Pregnant individuals, those with pre-existing neuromuscular and/or musculoskeletal dysfunctions prior to their professional nursing practice, and those undergoing continuous medication treatment and/or nonpharmacological therapy for chronic pain were excluded.

For characterization of the sample, demographic, anthropometric, and occupational data were collected. Demographic data included sex and age. The anthropometric evaluation consisted of measuring weight, height, and waist circumference (WC), as well as calculating the body mass index (BMI). Occupational data included professional category, department, shift, and length of employment. The perception and intensity of LBP were investigated using the Visual Analogue Scale (VAS), while occupational stress was assessed using the Job Stress Scale (JSS).

Participants were individually approached during their work shifts. The aim and importance of the study, data confidentiality, and instructions for filling out the questionnaires were explained, and once participation in the study was confirmed, a date for handing in and evaluating the questionnaires was scheduled. It should be noted that all questionnaires were self-administered by the participants.

The study was conducted in accordance with CNS resolution nº 466/12 and was approved by the Research Ethics Committee of Universidade de Santa Cruz do Sul (UNISC) under no. 1.851.758. All participants provided written informed consent.

### ANTHROPOMETRIC CHARACTERISTICS

Weight and stature were measured using a digital scale with a stadiometer (Welmy®, R-110 model, Brazil). Participants were placed in a standing position, barefoot, with feet together and their heads in the Frankfurt plane position. Weight and height measurements were used to calculate the BMI (weight [kg]/height [m]^2^), which was classified as follows: underweight (BMI < 18,5 kg/m2); normal weight (BMI ≥ 18,5 kg/m^2^ and ≤ 24,9 kg/m^2^); overweight (BMI ≥ 25 kg/m^2^ and ≤ 29,9 kg/m^2^); obese class I (BMI ≥ 30 kg/m^2^ and ≤ 34,9 kg/m^2^);obese class II (BMI ≥ 35 kg/m^2^ and ≤ 39,9 kg/m^2^); and obese class III (BMI 40 ≥ kg/m^2^).^17^ For statistical purposes, the underweight category was grouped together with normal range, while obesity classes I, II and III were grouped under obesity.

WC was measured at the narrowest wait, midway between the lowest rib and the iliac crest, using a nonstretching tape, as described by Lean et al.^18^ Measurements were classified as follows: normal risk (WC < 94 for men and < 80 cm for women); increased risk (WC ≥ 94 cm for men and ≥ 94 cm for women); and high risk (WC ≥ 102 cm for men and ≥ 88 cm for women).^17^

### PERCEPTION AND INTENSITY OF LBP

Perception and intensity of LBP were evaluated using the VAS, which is a tool consisting of an ordinal numeric scale ranging from 0 to 10 and figures of facial expressions that allow the person to identify and report the intensity of their perceived pain. The intensity was considered as follows: 0 points, total absence of pain; 1-2 points, slight pain; 3-7 points, moderate pain; 8-9 points, severe pain; and 10 points, maximum level of pain.^19^ Participants were assessed during their work shift, thus the perception and intensity of LBP reflects how they were feeling at the time of VAS application.

### OCCUPATIONAL STRESS

The JSS has been used to assess occupational stress and it provides sufficient data to classify participants according to the Karasek’s Demand-Control Model (DCM) of Job Stress, in which psychological demands refer to psychological matters and control demands refer to the possibility of using one’s intellectual skills during work activities.^20^ In our study, we used the validated Brazilian version of the JSS, which consists of 17 items distributed across three dimensions: (I) evaluation of psychological demands regarding time, deadlines, and conflicts during the execution of work activities, (II) evaluation of control at work regarding the use of skills and free will in decision-making, and (III) evaluation of social support regarding social relationships in the occupational setting.^20^ Only psychological and control demands were assessed for the application of DC.

According to the scores obtained in the demand and control dimensions, participants were classified as having high/low psychological demands and high/low control at work. The cut-off value adopted for high/low demand and high/low control was the median in each dimension. Individual participant scores were then compared with the medians. When the individual score was less than or equal to the median, the dimension was classified as low/low, and when the individual score was greater than the median, it was classified as high/high.^20,21^

Individual scores were combined to allow the reproduction of the four domains of occupational stress described in the DCM: (I) high strain (high psychological demand and low control at work), characterized as a psychological distress-inducing situation and, therefore, harmful – groups in this category are those most exposed to occupational stress; (II) active work (high psychological demand and high control at work), characterized by challenging situations that may promote professional growth; (III) passive work (low psychological demand and low control at work), characterized by disinterest due to a monotonous workplace – groups in this category have an intermediate exposure to occupational stress; and (IV) low strain (low psychological demand and high control at work) – this is considered the “ideal” situation and is associated with low risk for the onset of occupational illness.^20,21^

### STATISTICAL ANALYSIS

Data analyses were performed on SPSS 23.0. Data were expressed as frequency, measures of central tendency, and dispersion. The Shapiro-Wilk test was used to assess the normality of data, and Pearson’s chi-square test was used to assess the association between categorial variables. Statistical significance was set at p < 0.05.

## RESULTS

Of a total of 106 participants, 50,9% were aged ≤ 35 years, 40.6% were classified as overweight, and most were women (90.6%). Demographic, anthropometric, and occupational characteristics of the study sample are presented in Table 1.

**Table 1:** Descriptive characteristics of hospital nursing professionals (n =106)

Variables	n (%)
Sex	
Male	10 (9.4)
Female	96 (90.6)
Age (years)	
Up to 35	59 (50.9)
> 35	52 (49.1)
BMI	
Normal weight	42 (39.6)
Overweight	43 (40.6)
Obese	20 (18.9)
WC	
Normal risk	45 (42.5)
Increased risk	31 (29.2)
High risk	30 (28.3)
Professional category	
Nurse	11 (10.4)
Practical nurse	95 (89.6)
Department	
Ward	52 (49.0)
SU, OU, or ER	54 (51.0)
Work shift	
Day	71 (66.8)
Night	35 (33.2)
Length of employment (years)	
< 10	73 (68.9)
≥ 10	32 (30.1)

SU = surgical unit; WC = waist circumference; OU = obstetric unit; BMI = body mass index; ER = emergency room.

LBP prevalence was reported by 76.40% of participants (Table 2). Pain intensity was classified as “slight to moderate” by 79.01% of the sample, while 20.98% reported “intense” pain. Additionally, prevalence of low psychological demand (54.7%) and high control at work (63.2%) were observed in the JSS dimensions.

**Table 2:** Distribution of hospital nursing professionals according to low back pain (LBP) and the JSS dimensions (n = 106)

Variables	n (%)
Perception of LBP	
Presence	81 (76.4)
Absence	25 (23.6)
Intensity of LBP	
Total absence of pain	25 (23.6)
Slight to moderate	64 (60.4)
Severe	17 (16.0)
JSS	
Demand	
Low psychological demand	58 (54.7)
High psychological demand	48 (45.3)
Control	
Low control at work	39 (36.8)
High control at work	67 (63.2)

JSS = job stress scale.

**Figure 2:**
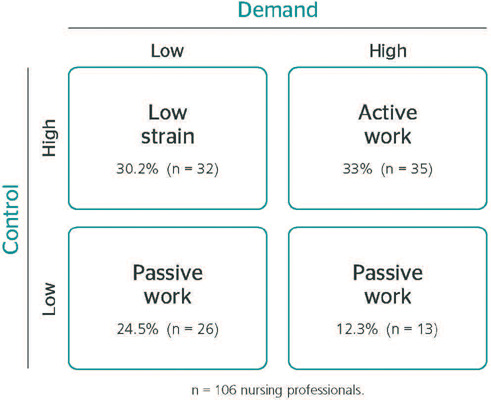
Distribution of nursing professionals according to the domains of occupational stress indicated in the demand-control model (D-C model).

According to the occupational stress domains described by the DCM (Figure 2), a higher prevalence of active work (high psychological demand and high control at work) and low strain (low psychological demand and high control at work) was observed in our sample, corresponding to 33.0% and 30.2%, respectively.

There were no statistically significant associations between the DCM domains of occupational stress and LBP presence (p= 0.721) (Table 3).

**Table 3:** Association between low back pain (LBP) and occupational stress among hospital nurses

Variables	Perception of LBP n (%)		p-value
	Presence (n = 81)	Absence (n = 25)	
DCM			
Low strain	23 (28.4)	9 (36.0)	0.721
Passive work	19 (23.4)	7 (28.0)	
Active work	29 (35.8)	6 (24.0)	
High strain	10 (12.3)	3 (12.0)	

Chi-square test; significance level at 5%.

DCM = Karasek’s demand-control model.

## DISCUSSION

This study assessed the association between LBP perception and occupational stress among nursing professionals in a teaching hospital located in southern Brazil. A high prevalence of LBP perception was found, but it was not significantly correlated with occupational stress in this study sample. The sample was mainly composed of women, with a high prevalence of overweight individuals. Studies demonstrate the predominance of women among nursing professionals, who are more likely to develop musculoskeletal symptoms and LBP.^14,22^ This reflects a historical characteristic of female predominance in the nursing profession, thus becoming relevant to consider the interaction between paid work and domestic activity as a potential exacerbating factor.^22^

Similarly, being overweight is an important risk factor for the development of LBP. A population-based study has shown that BMI can be an unfavorable indicator for the development of obesiuty.^22^ The association between these variables described in the literature may be explained by joint overload and changes in the gravitational axis, in which anti-gravitational actions overstrain muscles.^23^

The nursing staff is a frequent focus of research, especially in the hospital setting. The need for investigation arises from the adverse conditions associated with their activities, as well as exposure to different workloads^3,14^ when compared with other health care professionals.^4,5^ In our study, 30.2% of professionals have been working for ≥ 10 years.

According to Cargnin et al.,^9^ the duration of work constitutes an important risk factor for the occurrence of LBP. The authors demonstrated a significant relationship between the duration of work performed at angles greater than 45° of trunk flexion and the frequency and duration of LBP. They emphasize that physical and mental wear is cumulative and results from adopting inadequate postures for longer than 20 minutes.^9,24^

LBP is classified as an occupational disease and constitutes a global public health issue that requires prevention.^25^ There are multiple complex risk factors that interact with each other, and when related to professional practice, they involve work perception, task organization, and execution methods, as well as physical, psychosocial, and ergonomic demands.^26^ Our study showed a high prevalence of LBP (76.4%), while Shieh et al.^2^ and Jradi et al.^7^ found prevalences of 72% and 80% in samples of 788 and 427 hospital nursing professionals, respectively.

Sanjoy et al.^27^ highlight three occupational aspects associated with the presence of LBP in this professional category: (I) lack of support staff, (II) frequent manual lifting, and (III) overtime. Machado et al.^22^ emphasize the importance of expanding the number of professionals in the nursing staff, strengthening support networks, and adjusting work schedules, with the aim of optimizing the quality of care provided while contributing to the reduction of the risk of LBP development.

Regarding occupational stress, the DCM considers work activities performed under low work control and high psychological demand (high strain ) as detrimental to health.^13^ Our study showed a higher prevalence of individuals subjected to conditions of high control and high demand, as well as high control and low demand, corresponding to the categories of active work and low strain, respectively. The DCM characterizes active work as an indicator of intermediate risk for the development of occupational stress. Similarly, low job demand is considered an indicator of low risk of illness due to work-related causes.^13^

Cross-sectional studies have sought to describe this professional class in terms of occupational stress using the DCM. Braga et al.^26^ demonstrated, in a sample composed of 399 nursing professionals, the predominance of situations compatible with high control and high demand, which is classified as active work. Schmidt et al.^25^ investigated the relationship between occupational stress and job characteristics in 211 nursing professionals from the surgical staff, revealing a significant association between psychological demand and professional category (practical nurse or nurse), as well as between job control and the nature of the institution, professional category, and weekly workload. These authors emphasize the contribution of high psychological demand and low job control to the development of psychological distress and decline in quality of life, respectively.^25,26^

In addition to providing standard nursing care, nursing professionals also have to perform management activities, which can contribute to occupational overload.^25^ High job control was more prevalent in our study, and it can be seen as a compensatory mechanism against the deleterious effects of high psychological demand, contributing to the reduction of the risk of illness due to work-related causes.

Our sample had a high prevalence of LBP, with pain intensity ranging from slight to moderate, but it was not associated with the occupational stress domains described in the DCM. However, in Saudi Arabia and Ethiopia, Jradi et al.^7^ and Tefera et al.,^28^ respectively, assessed the psychosocial and occupational factors linked to the presence of LBP in nursing teams, identifying an association between LBP and work-related stress.

Although epidemiological data reveal a substantial increase in the prevalence of LBP among health care professionals, especially those working in nursing teams, and considering that this condition is one of the leading causes of disability, it is worth noting that there is a scarcity of reports on the association between LBP and psychosocial and occupational stress factors.^8^

This study has some limitations that need to be acknowledged, such as the collection of data at a single time point, imposed by the study design, as individuals with LBP may overestimate physical effort and/or psychosocial overload. In addition, there were no adjustments for occupational characteristics such as hospital department, which implies exposure to different levels of psychological demand and job control. Finally, the social support domain of the DCM was not included in the assessment of occupational stress in this study.

The present study has the peculiarity of evaluating the association of LBP perception with the domains of occupational stress described by the DCM, which is an important point to consider, given that occupational stressors are complex and interact with each other.

## CONCLUSIONS

The present study found a high prevalence of LBP, with pain intensity ranging from slight to moderate, that was not significantly associated with the domains of occupational stress described by the DCM in our sample of hospital nursing professionals. The implementation of preventive measures and interventions in the workplace can have direct implications on the design of public health policies to minimize the occurrence of LBP and occupational stress, both of which constitute serious public health issues. The assessment of occupational and psychological aspects associated with LBP in this professional category can contribute to the development of preventive and interventional actions, aiming primarily to alter modifiable risk factors and promote a better quality of life for these professionals.
